# Radiated radiofrequency immunity testing of automated external defibrillators - modifications of applicable standards are needed

**DOI:** 10.1186/1475-925X-10-66

**Published:** 2011-07-29

**Authors:** Ken Umberger, Howard I Bassen

**Affiliations:** 1 Fort Worth, TX 76052, USA; 2 Office of Science and Engineering Laboratories, U.S. Food and Drug Administration, 10903 New Hampshire Ave., Silver Spring MD, 20903, USA

## Abstract

**Background:**

We studied the worst-case radiated radiofrequency (RF) susceptibility of automated external defibrillators (AEDs) based on the electromagnetic compatibility (EMC) requirements of a current standard for cardiac defibrillators, IEC 60601-2-4. Square wave modulation was used to mimic cardiac physiological frequencies of 1 - 3 Hz. Deviations from the IEC standard were a lower frequency limit of 30 MHz to explore frequencies where the patient-connected leads could resonate. Also testing up to 20 V/m was performed. We tested AEDs with ventricular fibrillation (V-Fib) and normal sinus rhythm signals on the patient leads to enable testing for false negatives (inappropriate "no shock advised" by the AED).

**Methods:**

We performed radiated exposures in a 10 meter anechoic chamber using two broadband antennas to generate E fields in the 30 - 2500 MHz frequency range at 1% frequency steps. An AED patient simulator was housed in a shielded box and delivered normal and fibrillation waveforms to the AED's patient leads. We developed a technique to screen ECG waveforms stored in each AED for electromagnetic interference at all frequencies without waiting for the long cycle times between analyses (normally 20 to over 200 s).

**Results:**

Five of the seven AEDs tested were susceptible to RF interference, primarily at frequencies below 80 MHz. Some induced errors could cause AEDs to malfunction and effectively inhibit operator prompts to deliver a shock to a patient experiencing lethal fibrillation. Failures occurred in some AEDs exposed to E fields between 3 V/m and 20 V/m, in the 38 - 50 MHz range. These occurred when the patient simulator was delivering a V-Fib waveform to the AED. Also, we found it is not possible to test modern battery-only-operated AEDs for EMI using a patient simulator if the IEC 60601-2-4 defibrillator standard's simulated patient load is used.

**Conclusions:**

AEDs experienced potentially life-threatening false-negative failures from radiated RF, primarily below the lower frequency limit of present AED standards. Field strengths causing failures were at levels as low as 3 V/m at frequencies below 80 MHz where resonance of the patient leads and the AED input circuitry occurred. This plus problems with the standard's' prescribed patient load make changes to the standard necessary.

## Background

An AED is a portable, battery-powered electronic device that automatically diagnoses potentially life threatening irregular cardiac activity (arrhythmias) in a patient, such as ventricular fibrillation (V-Fib) and ventricular tachycardia (VT). Analysis of arrhythmias is done by monitoring the millivolt-level electrocardiographic (ECG) voltage on the patient's chest with two external electrodes (pads). The AED is able to identify and treat some arrhythmias by signaling the operator of the AED to initiate a high-voltage shock (therapy) to the patient by pressing one or more buttons. Ventricular fibrillation is a condition in which there is uncoordinated electrical propagation in the cardiac muscle of the ventricles. This chaotic electrical activity leads to inefficient contraction of the heart and a loss of blood flow to the brain and the rest of the body. Due to the lack of blood flow in the brain, irreversible brain damage and death can occur in just 5 minutes. Sudden cardiac arrest from V-Fib causes several hundred thousand deaths per year in the United States alone [1].

An AED's circuitry consists of three major subsystems: 1) Sensing, 2) Analysis and control, and 3) Shocking. The sensing subsystem contains the following to detect ECG data from the patient: two conductive wire leads attached to two separate electrodes (pads), analog electronics (usually consisting of a low-noise low-frequency amplifier and bandpass filter of less than 20 Hz), a high-voltage protection device, and an analog-to-digital (A-to-D) converter. The analysis and control subsystem contains signal processing hardware and software to analyze heart rhythm, a microprocessor to manage all operations of the device, memory for storage of data (ECG measurements, diagnosis and therapy delivery all vs. absolute time. Also included are a voice command generator, audio amplifier and speaker, and digital communications interface to download recorded data to a computer for display and printout of data. The shocking subsystem contains high-voltage charging circuitry, energy storage capacitors, and battery management and power conversion circuitry.

The following is the normal sequence of events that is performed automatically by typical AEDs once the electrodes are placed on the patient's chest. (1) A test is performed to determine if the electrodes were placed properly. If the impedance is not below around 200-300 Ω, the device prompts the operator to check the electrodes (pads). After a satisfactory pad check, the analysis begins. (2) ECG heart rhythm is analyzed for approximately 4 to 10 s; (3) The device decides if a shockable rhythm, i.e., VT or VF is present; (4) If a shock is advised, the high-voltage capacitor is charged; (5) A voice prompt is issued to the operator to push the shock button; (6) The operator must manually push a button on the AED to deliver a shock; (7) If the button is not pushed within a certain time frame, then the high-voltage capacitor is discharged automatically without shocking the patient; (8) If a non-shockable rhythm is detected, the AED issues voice prompt to perform CPR. This complete cycle can take from 20 s to over 2 minutes depending on the make and model of the AED.

### EMI issues motivating this study

We performed experimental electromagnetic interference (EMI) studies to test several commercially available AEDs for disruption of performance by radiated radiofrequency (RF) fields. This was done after learning of voluntary recalls by an AED manufacturer for EMI, after we could not obtain details about the interference. We considered the potential source of radiated RF susceptibility as being due to coupling of the E fields onto the unshielded patient-connected leads of AEDs. EMI pickup from leads and external wiring is recognized by other standards and experts in EMI/EMC [2-4]. These leads could couple radiated fields into the input circuits of an AED and cause EMI, potentially resulting in a malfunction.

Our initial goal was to perform standardized tests on commercially available AEDs for radiated RF interference. We wished to perform testing in accordance with AED-specific test methods and general medical device EMC standards. Once we began this testing it became clear that we needed to explore other parameters that affected the RF immunity of these devices. Also, it became clear that there were either omissions or problems with existing AEDs and medical device EMC standards that made worst-case testing of AEDs (with their long patient-connection leads) highly problematic at frequencies where we observed the greatest EM interference. The frequencies where worst-case conditions occurred were below 80 MHz.

### Relevant AED test standards

The primary EMC standards that apply to AEDs are in IEC 60601-2-4 [5] and IEC 60601-1-2 [6]. Both IEC 60601-1-2 and 60601-2-4 utilize the IEC's EMC radiated RF immunity test method standard 61000-4-3 [7]. The 61000-4-3 standard is for testing of equipment and is not specific to medical devices. We found significant problems attempting to comply with the test requirements of the IEC 60601-2-4 standard for testing modern AEDs [5]. None of the AEDs tested would operate (sense a simulated patient's cardiac electrical activity) under the simulated patient load requirements of the 60601-2-4 test standard. The EMC section of the IEC 60601-2-4 standard calls for radiated RF testing with no injection of simulated patient ECG signals waveforms on the patient leads (electrodes) during RF exposures. Also this EMC section calls for testing in the absence of noise (artifacts) such as those induced by cardiopulmonary resuscitation. The IEC standard for cardiac defibrillators (60601-2-4) defers to the medical device EMC standard IEC 60601-1-2-2007 for RF immunity testing below 80 MHz for defibrillators that are powered only by batteries. The 60601-1-2 standard requires testing for immunity to conducted disturbances, induced by RF fields. No radiated immunity testing is required below 80 MHz. The conducted immunity test method is specified IEC 61000-4-6 [8] for frequencies from 9 kHz to 80 MHz.

The simulated patient load specified in IEC 60601-2-4 presented problems. IEC 60601-2-4 specifies using 1 kΩ resistor in parallel with a 1 μF capacitor during radiated RF immunity tests. The problem we encountered was that none of the AEDs tested would recognize a 1 kΩ/1 μF load as a valid connection to a patient. The signal-input exemption, the battery-only exemption, and the simulated patient load problem required us to develop variations in the standardized test methods that could be used for AED testing to enable us to evaluate the worst-case RF immunity of these devices.

## Methods

### Overview

We studied seven commercially available AEDs to determine their susceptibility to radiated RF E fields over the frequency range 30 - 2500 MHz. We performed radiated exposures of AEDs in a "10 meter" fully anechoic chamber separately using two standard EMC antennas to generate 30 - 2500 MHz E fields. The AEDs were placed in a uniform test area at distances of 1.5 - 2 m from the antennas. The modifications mentioned above consisted of testing below the specified lower frequency limit of 80 MHz because we realized that radiated RF interference could occur in commercially available AEDs at frequencies from 30 to 60 MHz. A half wavelength at 80 MHz is 1.875 m and the leads were up to 132 cm each (2.64 m for the combined length of the two patient connected leads). We explored possible enhanced sensitivity due to the resonant length of the pair of leads or enhanced sensitivity due to electrically long leads resonating with the input reactance of the circuitry of the AED under test.

Another test modification we made was to use square-wave pulse-amplitude modulation (PAM) of the RF exposure signal at 1 and at 3 Hz. These two frequencies were chosen to approximately conform to the 2 Hz modulation frequency called for in the 60601-1-2 standard. Testing with both modulation frequencies was performed as follows. Once the worst-case RF carrier frequency was identified, both 1 and 3 Hz modulations were used sequentially to see which case was worse in terms of causing EM-induced failures. These modulations included the fundamental repetition frequency of normal cardiac waveforms (1-2 Hz). AEDs analyze the waveform present on their input (patient electrode leads) in order to determine if they should deliver a shock to a patient. NSR is a "healthy" ECG waveform. It has a fundamental frequency of 1-2 Hz but because of the sharp spiked nature of this natural ECG waveform, it contains many higher frequency harmonics. Ventricular tachycardia is a higher frequency waveform with a relatively strong amplitude but higher frequency than NSR. V-Fib waveforms from a patient contain significantly lower amplitudes and higher frequencies than the fundamental frequency of an NSR. We used 100% square wave amplitude modulation of our RF exposure field at frequencies of 1 to 4 Hz to stress the AED under test during our EMC testing. This is the approach specified in medical device EMC standards as discussed later in this paper.

### Exposure system

**Figure 1:**
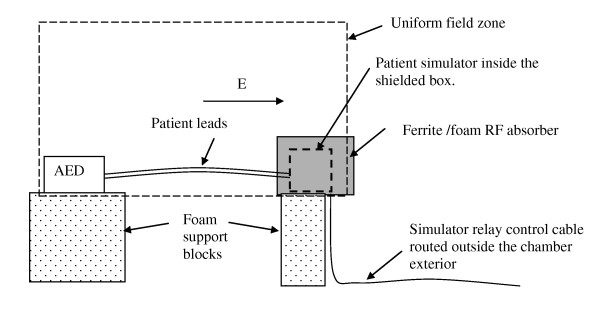
**Exposure system configuration (front view)**.

We designed a computer-automated exposure system in a "10 meter" fully anechoic chamber (TDK- Cedar Park, TX) and exposed devices using 1% frequency steps from 30 to 2500 MHz. Below 1000 MHz the field was uniform within 6 dB over a plane of 1.5 m × 1.5 m with no device under test present in the plane (Figure 1). The chamber was completely lined with ferrite tile absorber and covered with carbon-loaded plastic absorber (45 cm wedge construction). The bottom edge of the uniform field zone in this plane was 102 cm above the anechoic chamber's floor, and it was a vertical plane with an area of 1.5 m × 1.5 m as described in IEC 61000-4-3. We deviated from the IEC test method in order to produce a uniform field down to 30 MHz as follows: We used a chamber with an anechoic floor, rather than the conductive ground plane specified in the IEC standard. This did not affect the validity of our tests since we produced the same "uniform exposure field" that is specified in the IEC standard. This was verified by exposure field calibration (see below).

For the lower end of our test frequency range (30 - 100 MHz), we used a "biconilog" antenna (ETS model 3141, Austin, TX 78758 US). This antenna combined a log periodic antenna with a biconical dipole to provide broadband coverage from 30 - 1000 MHz. The lower frequency limit was chosen because our antenna and the amplifier that drove it were unable to operate below approximately 30 MHz. No other low frequency antennas were available that could provide both the required field strengths and the uniformity of the fields in our chamber. At frequencies below 100 MHz we used this antenna as a biconical dipole by orienting the "eggbeater" dipole elements closest to the AED and pointing the log periodic section away from the AED under test. From 100 - 1000 MHz the biconilog antenna was used in its normal configuration. From 1000 - 2500 MHz we used an ETS Lindgren model 3115 Double Ridge Horn antenna in its normal configuration to expose nine regions ("windows") to verify uniformity in the exposure plane. We used a synthesized signal generator (Agilent Digital RF Signal generator E4432B, Santa Clara CA 95051) controlled by a personal computer to produce the RF signal. The RF signals were amplified by the following devices. From 30 to 100 MHz we used an Instruments for Industry M406 Amplifier (Ronkonkoma N.Y.). From 100 to 400 MHz we used an ENI model 5100. From 400 to 1000 MHz we used an ENI model 6100 Amplifier (Rochester N.Y.). From 1000 to 2500 MHz we used an Ophir model 5163 Amplifier (Los Angeles, CA).

We developed custom EMC control software to semi-automatically perform all calibration and testing operations once the operating parameters were chosen by the operator. A laptop personal computer controlled the system hardware via custom software programmed in LabVIEW with a combination of GPIB and USB interfaces. The LabVIEW graphical user interface (GUI) provided control of the following parameters: signal power to the amplifier (and antenna), signal frequency, square-wave modulation frequency of the RF signal, E-field measurements from probes in the anechoic chamber, forward power, reflected power, and if needed, digital oscilloscope measurements.

### Exposure field calibration and generation

From 30 to 1000 MHz a field calibration was performed to confirm a uniform E field level at all frequencies and at all 16 points in the exposure area. Above 1000 MHz we measured points in 9 locations, each 0.5 × 0.5 m in the 1.5 m × 1.5 m plane. Frequency was stepped in 1% increments. The maximum variation for the E field at any of the 16 points was 6 dB at each frequency. This was measured with isotropic E field probes (ETS-Lindgren HI-6105, Cedar Park, TX 78613 US) linked via fiber optics to a remote field strength readout from ETS-Lindgren (model HI-6100) outside the anechoic chamber. The probe was placed individually at each of 16 points (9 points for 1000-2500 MHz) and readings were taken of the E field components at each of the 450 frequencies from 30 - 2500 MHz. Horizontal polarization was used for most exposures to achieve the maximum effect of interference to the horizontally oriented patient leads. The E field level in the exposure area was adjusted at each frequency to the desired level by the software controlling the signal generator output level.

### AED patient simulator

We designed an RF-compatible patient simulator (AED tester) to deliver normal and fibrillation waveforms to the AED during RF exposure testing. We used two Delta 1500 Automated External Defibrillator Analyzers connected in a special configuration (Netech Corporation 110 Toledo Street, Farmingdale, NY 11735 U.S.) applying various ECG waveforms to the AED under test. This analyzer/tester can generate three standard ECG waveforms at amplitudes identical to those from actual patients. Its low output impedance (50 Ω) is designed to deliver the same voltage to the sensing circuitry of an AED as the case when the AED is connected to a person being assessed for V-Fib.

**Figure 2:**
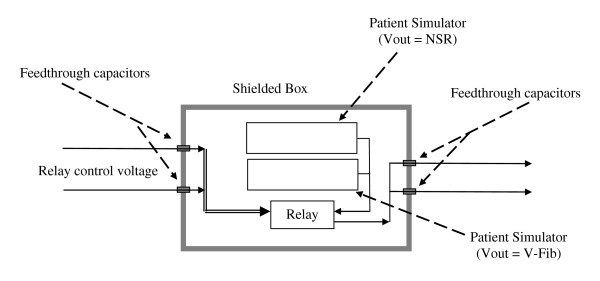
**RF-compatible patient simulator**.

The waveforms are NSR, ventricular tachycardia (VT), and VF. One Delta Analyzer was set to continuously output an NSR waveform while the other Delta Analyzer was set to output a V-fib waveform. We used an electromechanical relay to remotely switch one of the two Delta Analyzer outputs to the leads of the AED under test. The two Delta Analyzers and the relay were mounted inside a metallic shielded box (35 cm × 30 cm × 6 cm) with a continuous-hinged door to minimize interference of the Delta Analyzers from radiated RF fields. The relay was switched remotely by applying a DC voltage via a shielded cable that was oriented perpendicular to the exposure E field. The AED leads were terminated in a 50 Ω resistor that was mounted immediately outside the shielded box. All connections into and out of the shielded box were filtered through 5000 pF high-voltage feedthrough capacitors. The shielded box was placed behind a panel of ferrite anechoic absorber (61 × 61 cm) to minimize reflections from the box. The entire setup is shown in Figure 2. The entire system was tested and found not to alter the field uniformity over the frequency range.

### Audio prompt monitoring

During the exposure, audio from voice prompts generated by the AED were monitored acoustically with a plastic funnel placed approximately 100 cm from the AED. The funnel neck was attached to a rubber hose that was routed out of the anechoic chamber and into a small microphone. This avoided EMI in the microphone, its cables, and the audio amplifier fed by the microphone. The AED's voice commands such as "analyzing heart rhythm", "waiting for shock", "shock advised", and "start CPR" were used by test personnel to start RF exposure during the analysis mode or to hear warnings (e.g., "check the pads", "motion detected").

### Devices tested

Seven different models of AEDs were purchased either new or used. Their relatively low cost ($1000 - $2000) when new would make them likely candidates for home and Public Access Defibrillator (PAD) use. Each was from a different manufacturer, providing a cross section of device designs for testing. All were battery powered with no provision for external power connections. All devices issued audio prompts to the operator and some had a text message or lights on the panel to display instructions to the operator for the next step to perform, such as a flashing light to indicate the button to push to initiate a shock or lights that highlighted a graphic on the face of the device to identify the action expected by the operator. The devices tested are listed in Table 1 with the manufacturers' names omitted. The CPR cycle times are listed to show how much time it adds to the total operating cycle from analysis to CPR and back to analysis again. The lead length is also listed to provide a reference point for considering wavelength resonance (as discussed in the results section).

**Table 1:** Devices tested: lead length and CPR cycle time

Device ID	Lead length (cm)	CPR cycle time (s)
AED1	115	20

AED2	95	> 120

AED3	101	20

AED4	132	60

AED5	128	90

AED6	115	20

AED7	118	60

### Radiated RF immunity testing

Radiated RF immunity testing was performed on each AED in the uniform field area (horizontal polarization) with its patient leads stretched horizontally and connected to the patient simulator inside the shielded box. Testing was done over the 30 - 2500 MHz range at 1% steps requiring over 450 test frequencies. We exposed each AED under test to each of the test frequencies for only 1 - 3 s (dwell time) before going on to the next frequency during the "screening test." This special test (described in more detail below) used unique methods to evaluate the AED's instantaneous response to RF voltages induced on its ECG monitoring circuitry. If we exposed each of the AEDs to each test frequency during the AED's analyze phase (the most critical function of an AED) and waited for a response, a much more lengthy series of tests would be required. By using the screening test we avoided testing during the long period that AEDs require to make a shock decision (20 - 200 s or longer). Consideration of battery life and the volume of data to analyze for EMI led to the decision to perform the "screening test". This test identified an AED's susceptibility to EMI in terms of RF frequencies and amplitude modulations. The screening test for each AED involved stepping through the full frequency range (using square-wave-modulated RF) and dwelling 1 to 3 s per step while the device cycled through all modes. AED reactions to RF were not monitored by wires. They were monitored by examining stored ECG data after the AED was exposed to a sequence of all RF frequencies and it was removed from the anechoic chamber. The AED's internal software stored ECG waveforms detected by the AED's leads, the time of day, any diagnostic and test data including records of operator-warning messages that were sent to the voice output system of the AED. After exposure was completed and the AED was removed from the anechoic chamber, the stored ECG was downloaded to another computer and printed out for manual analysis.

In addition to the data recorded from the AED under test, we recorded a time stamp in the RF exposure data. This was done via software in the RF exposure system that controlled the RF signal that was sent to the antenna in the anechoic chamber. This tagged each exposure frequency step with a time of day. This was used to correlate the exposure field's RF frequency to the time of day on the ECG record. This required that the time in the exposure computer and the AED under test be synchronized before starting testing. All the AEDs resolved time to at least one second; however, some could not be set as accurately to the same absolute time as the RF exposure control computer. One particular AED was very difficult to set to absolute time better than to the nearest minute. In that particular AED, we started exposing at a frequency know to generate "noise" (distortion) that was visible on the ECG (possible interference). The start of "noise" was used to synchronize the data. Another AED only recorded elapsed time from power ON of the device. For that unit, we used a feature in the software controlling the RF exposure computer. This feature was a start test button that allowed us to synchronize the RF exposure data to the elapsed time recording of the AED.

**Figure 3:**
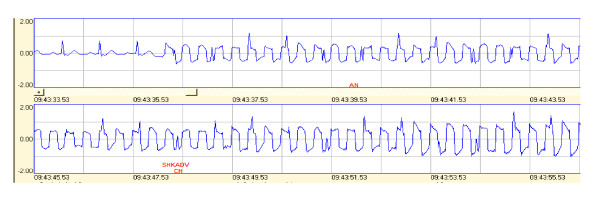
**Failures as seen in stored ECG records in a short segment of ECG recordings**. Recording from AED2 while analyzing an NSR input from the patient simulator. Exposure to 10 V/m with 3 Hz pulsed modulation. Frequency is stepped from 42 MHz at start (top left) to 47 MHz at end of the recording (bottom right). Shock was advised. NSR is not distorted for the first 3 cycles.

After synchronization of the time stamps on the AED data, the ECG record was analyzed for any anomalies in the recorded waveform indicating interference from the pulsed RF. For a first pass using screening-test data, we looked for qualitative changes in the spiked shape of a clean NSR waveform fed from the patient simulator (Figure 3) vs. the square wave shape of an EMI-induced distortion. The time stamps were compared to determine what frequency of interference was showing up in the ECG. Frequencies were marked on the printed ECG and relative amplitude of the ECG data was noted. In order to identify potential EMI by reviewing the recorded ECG waveforms, we looked for qualitative changes in the spiked shape of a clean NSR waveform. This clean waveform was fed from the patient simulator (Figure 3). It is distinct from the square wave shape of an EMI-induced distortion in the ECG. The square wave shape is due to the pulsed modulation we imposed on the RF field. As seen in Figure 3, changing the RF exposure frequency slightly created a significant increase or decrease in the recorded ECG shape. This indicated the probable presence or absence of EMI. Later we used longer durations to expose AEDs at the particular frequencies identified in the screening test as probably causing EMI.

This was done to rapidly identify the frequencies that were likely to cause EMI .at field strengths of as low as 3 V/m up to 20 V/m. The range of frequencies where the interfering RF was measureable in the ECG was noted for retesting later. This screening test drastically reduced test time yet identified most possible EMI problems. Later, testing in a narrow frequency range or at single frequencies was performed while the AED under test was in the analysis mode since that is the time a decision is made concerning shock therapy.

During exposure, the patient simulator was set to deliver an ECG waveform (NSR or V-Fib) depending on the modulation frequency imposed on the RF exposure field. For example, if NSR was being applied, a square-wave modulation frequency of 3 - 4 Hz was used to simulate fibrillation. If V-Fib was delivered by the patient simulator, then 1 Hz square-wave modulation was used to simulate a normal 60 beats per minute heart rhythm. We did not test AEDs with the patient simulator set to generate VTAC waveforms. We did not have the resources to perform our detailed protocol for this "intermediate" risk condition on seven AEDs, each tested at over 450 frequencies. We believe that testing for the two bounding conditions (worst-case/best case) of V-Fib and NSR were sufficient to identify EMI vulnerabilities of each AED.

### EMI failure conditions studied

During the analysis and pre-shock stages of operation we looked at the stored ECG data after exposure for the following problems. All of these conditions are serious, either resulting in no resuscitation of the patient or in the delivery of a shock when not warranted. Krauthamer [9] defines "false positive" and "false negative" outcomes for AEDs as follows. False Negative is defined as no shock advised when a patient has a "shockable rhythm" (i.e. V-Fib), and False Positive condition occurred if a Normal Sinus Rhythm (NSR) was present at the patient electrodes, but EMI caused the AED to advise a shock to be delivered. For our tests the following were observed. A False Negative condition occurred when V-Fib was present but not recognized by an AED and no shock was advised. A False Positive occurred when NSR was applied to the patient electrodes, but interference from 3 Hz pulsed RF caused the AED to recommend "shock advised". We tested AEDs with the patient simulator set to generate V-Fib, and in a separate test, with the simulator set to generate NSR waveforms.

Another common type of false negative condition was when the AED incorrectly identified EMI as patient movement or poor electrode contact with the patient. Then the AED would not analyze the patient's condition while interference was present, preventing a shock to be advised if V-Fib were to be present.

## Results

The screening test provided valuable information. As seen in Figure 3, changing the RF exposure frequency slightly resulted in a significant increase or decrease in the recorded ECG shape. This indicated the probable presence or absence of EMI. Later we used longer durations to expose at certain frequencies identified as probably causing EMI. Little interference was seen for most AEDs above 80 MHz because of the RF voltages on the input of the AED under test were not sufficiently large enough to induce interference. RF voltages induced on a pair of wires (the patient leads) by a uniform electric field are independent of frequency except as follows. At certain frequencies we observed interference in certain AEDs. This interference was observed in the form of distortion (noise) in the stored ECG waveforms at certain exposure frequencies. This was due to increased RF voltage pickup in the AED input circuitry. This increased pickup is believed to be due to resonances of the leads in combination with the AED input impedance. The stored ECG data recorded during exposure to "non resonant" frequencies provided a baseline to compare with frequencies where distortion occurred due to higher RF voltages induced into the AED.

We exposed devices at their most sensitive frequencies as determined in the screening tests to see how the RF affected the decision mode of an AED. It was expected that problems would occur primarily during the analysis mode. The effects we evaluated are listed above in the section on EMI failure conditions studied. This was intended to see if an AED would accurately analyze the heart rhythm and correctly advise either to shock or not to shock based on the waveform it was processing. The AEDs had the following problematic responses to radiated RF exposure during screening tests and/or full tests with both V-Fib and NSR waveforms from the patient simulator applied to the AED. False positive - when NSR was applied by the patient simulator and during RF exposure, the AED advised to shock patient. False negative - when V-Fib was applied by the patient simulator and during RF exposure, the AED did not advise to shock the patient. Other failures resulted in halting of an analysis, in turn resulting in false negative or false positive responses, depending on the input waveform. Voice-prompt responses included: "Pads not connected", "check pads", "connect electrodes to patient", "Motion detected", "stop moving the patient", and "interference detected". Each of these indicated that the AED was seeing interference of some sort and could not determine if the electrodes were attached to a patient. This caused the AED to halt or delay analysis until cessation of EM exposure. This would have resulted in a delay or lack of therapy to the patent, which could have potentially fatal effects.

**Figure 4:**
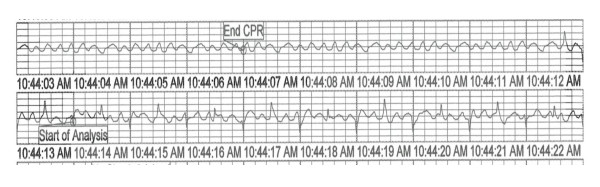
**Stored electrogram of AED4 while analyzing a V-Fib input from the patient simulator**. RF is stepped from 42 MHz (top left) to 44 MHz (bottom right) at 15 V/m, with 1 Hz pulsed RF. No shock was advised.

The failures discovered when we performed screening tests (post-exposure evaluation of stored ECG and other records) as well as failures during real-time audio monitoring are presented in Tables 2, 3, 4, 5, 6, 7, &8 Figure 3 and 4. Note that AED2's failure at the low value of 3 V/m highlights the error of the allowances specified in IEC 60601-2-4. Also, 60601-1-2 specifies a radiated RF immunity of 10 V/m for life-supporting devices.

**Table 2:** AED1 tests results

Device designation	Type of interference observed	Field strength for worst-case failure (V/m)	Modulation(Hz)	Frequency range of failure (MHz)	Device's behavior (Observations from user standpoint)	Notes
**AED1**	False negative	13	1 Hz pulsed (square wave)	39-44	Verbal response to fibrillation: "No shock advised"	V-Fib applied - no shock advised. Interference caused distorted ECG; analyzed as NSR. Analysis delayed during RF exposure.

**AED1**	False positive	20	4 Hz pulsed (square wave)	50 (single frequency test)	After analysis AED audio prompt: "Shock advised" and "shock advised" appeared in the ECG printout	NSR applied but shock advised. Interference masked the normal heart rhythm at a higher frequency

**Table 3:** AED 2 test results

Device designation	Type of interference observed	Field strength for worst-case failure (V/m)	Modulation (Hz)	Frequency range of failure (MHz)	Device's behavior (Observations from user standpoint)	Notes
**AED2**	False negative	3	1 Hz pulsed (square wave)	43-46	Verbal response after analyze mode: "Interference detected" then "No shock advised"	V-Fib applied - No shock advised Interference 8× higher amplitude than normal ECG

**AED2**	False negative	20	1 Hz pulsed (square wave)	300-322; 366-370, and 900-915	Buzz was heard in the audio from the device.	V-Fib applied - voice prompts interfered by buzzing sound

**AED2**	False positive	4	3 Hz pulsed (square wave)	31-34 42-47	Verbal warning after analyzing heart rhythm: "Shock advised" at 47-49 MHz	NSR applied -"shock advised" RF caused spikes in ECG. Interpreted as fibrillation. Shock canceled at 34 MHz

**Table 4:** AED3 test results

Device designation	Type of interference observed	Field strength for worst-case failure (V/m)	Modulation	Frequency range of failure (MHz)	Device's behavior (Observations from user standpoint)	Notes
**AED3**	False negative	20	1 Hz pulsed (square wave)	40-44	Verbal warnings: "Check pads" always resulted in delayed analysis.	VF applied - check pads warning Distortion in recorded ECG

**AED3**	False negative	20	1 Hz pulsed (square wave)	30-80	Verbal warning from device: "Press pads firmly to patient's bare skin"	Distortion in recorded ECG

**Table 5:** AED4 test results

Device designation	Type of interference observed	Field strength for worst-case failure (V/m)	Modulation (Hz)	Frequency range of failure (MHz)	Device's behavior (Observations from user standpoint)	Notes
**AED4**	None	20	1 Hz pulsed (square wave)	No failures	No verbal warnings heard during testing at any frequency. Device recommended shock as expected.	V-Fib applied - No interference or waveform distortion in ECG.

**Table 6:** AED5 test results

Device designation	Type of interference observed	Field strength for worst-case failure (V/m)	Modulation (Hz)	Frequency range of failure (MHz)	Device's behavior (Observations from user standpoint)	Notes
**AED5**	False negative	20 V/m	1 Hz pulsed (square wave)	38-41 and 47-49	Verbal warning heard during RF exposure while in analyze mode: "Stop all motion", "No one should touch the patient" "shock not advised"	V-Fib applied - no shock advised. No waveform distortion noted in the recorded waveforms.

**Table 7:** AED6 test results

Device designation	Type of interference observed	Field strength for worst-case failure (V/m)	Modulation (Hz)	Frequency range of failure (MHz)	Device's behavior (Observations from user standpoint)	Notes
**AED6**	No failures	20	1 Hz pulsed (square wave)	None	No verbal warnings heard during testing at any frequency.	This device only provides short ECG records. Insufficient data to fully evaluate ECG.

**Table 8:** AED7 test results

Device designation	Type of interference observed	Field strength for worst-case failure (V/m)	Modulation (Hz)	Frequency range of failure (MHz)	Device's behavior (Observations from user standpoint)	Notes
**AED7**	False negative	16	1 Hz pulsed (square wave)	38-43	Voice prompt- "check electrode pads". Flat line for ECG displayed on LCD;	V-Fib Applied -No shock advised ECG printout garbled "Pads off" annotation

## Discussion

We identified several technical limitations or issues in the requirements or test methods of present standards for EMC testing of AEDs. These problems involve allowances from conducted RF immunity testing at frequencies below 80 MHz as well as errors or omissions involving a patient simulator. These problems result in inadequate RF immunity testing of AEDs. The existing AED test standard [5] has no requirement for use of a patient simulator to deliver an ECG voltage to the AED under test while undergoing EMC testing. This does not allow testing for proper operation of the AED (detection of NSR or V-fib and preparation to deliver a shock).

One problem is that the simulated patient load specified in IEC 60601-2-4 for use during radiated RF immunity tests is a 1 kΩ resistor in parallel with a 1 μF capacitor. The problem we encountered was that none of the AEDs tested would recognize a 1 kΩ/1 μF load as a valid connection to a patient. Consequently, the AEDs would continue prompting the operator to attach the electrodes and would not operate properly. For this reason we used our AED patient simulator's internal 50 Ω load to allow the device to function normally. Therefore, we recommend changing the test requirement for radiated EMC immunity testing to require a 50 Ω load across the patient-connected electrodes plus the use of a patient simulator that delivers simulated cardiac electrical signals to the AED.

Another issue is the use of radiated vs. conducted immunity testing. Each of the patient leads of the AEDs we tested had lengths ranging from 90 to 132 cm. We found that this caused resonant conditions that made the AEDs most susceptible at frequencies below 80 MHz. Therefore we explored radiated immunity to frequencies as low as 30 MHz based on limits of our equipment. We exposed the device and its leads in a radiated field, aligned with the horizontally polarized electric field. Worst-case interference occurred below 80 MHz. The IEC standards 60601-1-2 and 60601-2-4 do not require radiated RF immunity testing below 80 MHz. Instead, direct injection testing is specified for these lower frequencies, coupling RF voltage and current onto cables or wires of the device under test. This is done using coupling networks or "coupling clamps" [8] that inject signals into the leads by inductive, capacitive, or resistive means. The IEC 60601-2-4 standard calls for this type of test but only onto the input power cord. But, since AEDs are all battery powered and do not have external power input, they are excluded from conducted RF immunity testing below 80 MHz. We think that it is imperative that AEDs be tested for conducted RF immunity near the resonant frequencies of the patient-connected leads. We also think that correlation between radiated and conducted RF immunity testing should be established for these devices below 80 MHz.

We also developed a screening method to accelerate initial testing of AEDs by not requiring exposure of the AED under test for the entire duration of the lengthy analysis cycle of the AED. This analysis cycle is typically 20 - 200 s. We exposed each AED to 450 frequencies, but only for less than 3 s at each frequency, thereby reducing the test cycle for a single AED to a few hours instead of many hours or even days. This was done by reviewing stored ECG data for the presence of interference, after the test exposure was competed. The interfering frequencies were identified by correlating the time of day stamp on the ECG with the time of day data for RF frequencies in the control computer.

Regarding amplitude modulation of the RF signal that exposed the AEDs under test, the IEC 60601-1-2 standard (medical device EMC) requires 2 Hz sinusoidal modulation or the most relevant modulation for a device with specific vulnerable frequencies. The 60601-2-4 AED standard requires 5 Hz modulation in its EMC section. We used 100% amplitude modulation in the form of pulses rather than sinusoidal signals. These have more harmonic content, which is more likely to cause interference than sinusoidal AM. Pulse modulation also subjects AEDs to a more severe test, simulating newer digital modulation schemes, as is done in other cardiac device EMI tests [10] and other non-medical EMI standards [2]. We used 1 - 4 Hz modulation in the preliminary tests. When we saw interference effects with 1 Hz and 3 Hz signals, we decided to use these in our final tests. Our use of 1 Hz is a good test of an AED's correct response to NSR (1 Hz). The 5 Hz AM modulation requirement of the 60601-2-4 standard was not used. The 3 Hz square wave modulation contains substantial 6 Hz and higher frequency harmonics that we found induced more failures related to V-Fib than modulation at higher frequencies.

## Conclusions

During testing of seven AEDs from each of the major manufacturers of AEDs sold in the U.S. we identified limitations with the applicable international standards and potential problems with the devices. After we corrected the problem for EMI testing by using a 50 Ω patient simulating load impedance (instead of the 1 kΩ/1 μF impedance) we found that all AEDs were operational, but most AEDs were susceptible to radiated RF interference at certain frequencies and modulations. Interference occurred primarily in the 30 - 80 MHz range with the RF signal amplitude modulated at 1 and 3 Hz with pulses. The RF field strengths causing interference ranged from 3 to 20 V/m. This was problematic with respect to the existing IEC medical device EMC and defibrillator standards. This is because they exclude any and all EMC testing of battery-only operated AEDs at frequencies below 80 MHz. Therefore, we recommend that modifications be made to the IEC 60601-2-4 cardiac defibrillator standard to correct two problems we encountered.

First, we recommend that for EMC testing with radiated RF electromagnetic fields, the patient leads of the AED under test should be connected to an active patient simulator with a patient load impedance of 50 Ω rather than 1 kΩ/1 μF specified in IEC 60601-2-4 standard. A patient simulator should deliver simulated V-Fib and NSR waveforms to the AED's patient leads. Secondly, we recommend that in addition to conducted immunity testing below 80 MHz, radiated RF immunity testing of AEDs should be performed with the patient-connected leads fully extended and aligned with the incident E-field. This radiated immunity testing should be done with a lower limit of 30 MHz rather than 80 MHz.

Much attention is already given to increasing the upper frequency limit of radiated RF immunity testing due to increased spectrum utilization in those ranges. The same attention should be given to the lower frequencies (27 - 80 MHz) for RF immunity due to the effects we observed, plus the prevalence of sources in this range. Sources in this range include hand-held amateur (HAM) radios authorized for use at 50 MHz, and the Industrial Scientific and Medical (ISM) bands at 27.12 MHz and 40.68 MHz for sources in the US and elsewhere. Radio Controlled Vehicles such as model airplanes and cars utilize handheld transmitters in the 27 MHz and 49 - 50 MHz bands. Certain marine band radios operate below 50 MHz. In addition, new EM emitters are constantly emerging as the RF spectrum becomes more crowded and the lower frequency range might become more commonly used, especially in the minimally-regulated ISM bands.

In the future we intend to replace our patient simulator that is controlled by wires with a saline patient simulator with non-metallic fiber-optically linked control cables. The simulator should generate either an NSR or a V-Fib waveform that can be remotely switched while RF exposure is performed. This will further improve the accuracy of our measurements of the effects of the leads and the body of a patient on EMI of an AED. Injected (conducted) RF immunity testing below 80 MHz should be explored for correlation with radiated RF immunity testing.

## Competing interests

The authors declare that they have no competing interests.

The mention of commercial products, their sources, or their use in connection with material reported herein is not to be construed as either an actual or implied endorsement of such products by the U.S. Department of Health and Human Services.

## Authors' contributions

KU planned and carried out the measurements, participated in the data analysis and drafted the manuscript. HB conceived of the study, planned the measurements, participated in the study's design and shared in drafting the manuscript. All authors read and approved the final manuscript.

## References

[B1] Zipes D Hein J Wellins J Sudden Cardiac Death Circulation 1998 98 2334 2351 9826323 10.1161/01.cir.98.21.2334

[B2] MIL-STD 461F DOD Requirements for the control of electromagnetic interference characteristics of subsystems and equipment 2007 U.S Dept. of Defense

[B3] Bassen H Ruggera P Casamento J Changes In The Susceptibility Of A Medical Device Resulting From Connection To A Full-Size Model Of A Human Proc of the Annual International Conference of the IEEE Engineering in Medicine and Biology Society 1992 14 2832 2834

[B4] Weston D Electromagnetic Compatibility 2001 Second Marcel Dekker

[B5] IEC 60601-2-4:2010 Medical electrical equipment - Part 2-4: Particular requirements for the basic safety and essential performance of cardiac defibrillators International Electrotechnical Commission 2010

[B6] IEC 60601-1-2:2007 Medical electrical equipment -- Part 1-2: General requirements for basic safety and essential performance -- Collateral standard: Electromagnetic compatibility -- Requirements and tests International Electrotechnical Commission 2007

[B7] IEC 61000-4-3:2008 Electromagnetic compatibility (EMC) - Part 4-3: Testing and measurement techniques - Radiated, radio-frequency, electromagnetic field immunity test International Electrotechnical Commission 2008

[B8] IEC 61000-4-6:2008 Electromagnetic compatibility (EMC) - Part 4-6: Testing and measurement techniques - Immunity to conducted disturbances, induced by radio-frequency fields International Electrotechnical Commission 2008

[B9] Krauthamer V Aspects of Automated Rhythm Detection Capabilities for AEDs EP Lab Digest 2010 1 1 38 40

[B10] ANSI/AAMI PC69:2007 Active implantable medical devices -- Electromagnetic compatibility -- EMC test protocols for implantable cardiac pacemakers and implantable cardioverter defibrillators Association for the Advancement of Medical Instrumentation Approved 12 April 2007 by American National Standards Institute, Inc

